# Urbanization impact on mosquito community and the transmission potential of filarial infection in central Europe

**DOI:** 10.1186/s13071-018-2845-1

**Published:** 2018-04-24

**Authors:** Viktória Čabanová, Martina Miterpáková, Daniela Valentová, Hana Blažejová, Ivo Rudolf, Eduard Stloukal, Zuzana Hurníková, Marianna Dzidová

**Affiliations:** 1 Institute of Parasitology, Slovak Academy of Sciences, Hlinkova 3, 040 01 Košice, Slovakia; 2 State Veterinary and Food Institute, Botanická 15, 842 52 Bratislava, Slovakia; 3 Institute of Vertebrate Biology, v.v.i, Czech Academy of Sciences, Květná 8, 603 65 Brno, Czech Republic; 4 Department of Zoology, Faculty of Natural Sciences, Comenius University, Mlynská dolina B-1, SK-842 15 Bratislava, Slovakia

**Keywords:** *Dirofilaria*, Mosquito-borne diseases, *Culex pipiens* complex, *Anopheles maculipennis* complex, Xenomonitoring

## Abstract

**Background:**

Despite long-term research on dirofilariosis in Slovakia, little attention has thus far been paid to *Dirofilaria* vectors. The particular aim of the present study was molecular screening for filarioid parasites in two different habitats of Bratislava, the capital city of Slovakia. In addition, the effect of urbanisation on mosquito species abundance and composition, associated with the risk of mosquito-borne infections, was studied and discussed.

**Methods:**

Mosquitoes were identified by morphological features, and molecular methods were also used for determination of selected individuals belonging to cryptic species from the *Anopheles maculipennis* and *Culex pipiens* complexes. The presence of filarioid DNA (*Dirofilaria repens*, *Dirofilaria immitis* and *Setaria* spp.) was detected using standard PCR approaches and sequencing.

**Results:**

A total of 6957 female mosquitoes were collected for the study. Overall, the most abundant mosquito species was *Aedes vexans*, closely followed by unidentified members of the *Cx. pipiens* complex and the less numerous but still plentiful *Ochlerotatus sticticus* species. Further investigation of mosquito material revealed 4.26% relative prevalence of *Dirofilaria* spp., whereby both species, *D. repens* and *D. immitis*, were identified. The majority of positive mosquito pools had their origin in a floodplain area on the outskirts of the city, with a relative prevalence of 5.32%; only two mosquito pools (1.26%) were shown to be positive in the residential zone of Bratislava. *Setaria* spp. DNA was not detected in mosquitoes within this study.

**Conclusions:**

The study presented herein represents initial research focused on molecular mosquito screening for filarioid parasites in urban and urban-fringe habitats of Bratislava, Slovakia. Molecular analyses within the *Cx. pipiens* complex identified two biotypes: *Cx. pipiens* biotype *pipiens* and *Cx. pipiens* biotype *molestus*. To our knowledge, *Dirofilaria* spp. were detected for the first time in Slovakia in mosquitoes other than *Ae. vexans*, i.e. *D. repens* in *Anopheles messeae* and unidentified members of *An. maculipennis* and *Cx. pipiens* complexes, and *D. immitis* in *Coquillettidia richiardii* and *Cx. pipiens* biotype *pipiens.* Both dirofilarial species were found in *Och. sticticus.* The suitable conditions for the vectors’ biology would represent the main risk factor for dirofilariosis transmission.

## Background

In recent years, increasing attention has been focused on mosquitoes worldwide due to the growing spread of invasive mosquito species as well as mosquito-borne infectious diseases [1]. Besides viruses, mosquitoes are also responsible for the transmission of medically important protozoans and nematodes, such as the *Plasomodium* species that causes malaria and several filarioid parasites of human and animals, including *Wuchereria bancrofti*, *Brugia* spp. or *Dirofilaria* spp. [2, 3]. In Europe, dirofilariosis is currently of medical importance. Pets, such as dogs and cats, are the most frequently affected with *D. repens* and *D. immitis*, while human infections are primarily caused by *D. repens*, which is responsible for the subcutaneous and ocular form of the disease [4].

Different mosquito species, mainly of the genera *Aedes*/*Ochlerotatus*, *Anopheles* and *Culex*, are implicated in *Dirofilaria* spp. transmission [5]. Given the fact that mosquitoes and developing *Dirofilaria* larvae are strictly dependent on climatic factors, mainly air temperature, the current global environmental change has led to an increase in the spreading of the parasite from endemic areas to previously non-affected regions. Moreover, climate-dependent introduction of invasive mosquito species as well as the spread of exotic mosquitoes (such as *Aedes albopictus*, *Aedes japonicus* and *Aedes koreicus*) through commercial activities are also of a great importance in dirofilariosis (and other mosquito-borne diseases) expansion [2, 6]. At present, the effect of landscape anthropization on mosquito populations is a key factor in the spreading of vector-borne pathogens among both pets and the human communities [7].

Slovakia, located in central Europe, was reported to be endemic for canine dirofilariosis caused by *D. repens* approximately ten years ago [8]. Since then, the detailed results of a comprehensive epidemiological study have been published [9] and several human cases of autochthonous dirofilariosis described [10]. Despite the long-term research, little attention has thus far been paid to *Dirofilaria* vectors. The first screening for dirofilariosis in mosquitoes was performed in 2013 and showed that the species *Aedes vexans* was incorporated into both the *D. repens* and the *D. immitis* life-cycles in Slovakia [11, 12]. However, the mentioned screening was restricted only to several locations in the Košice region, in the south-eastern part of the country. Because most *Dirofilaria*-infected people in Slovakia come from the south-western part of the country bordering Austria and Hungary, which is also considered endemic area for canine dirofilariosis, we decided to conduct our initial research in this region. The particular aim of the present study was molecular screening for filarioid parasites in mosquitoes collected in two different habitats in Bratislava, the capital city of Slovakia. In addition, the effect of urbanisation on mosquito species abundance and composition associated with the assessment of the risk of mosquito-borne infections was studied and discussed.

## Methods

### Studied localities

**Fig. 1:**
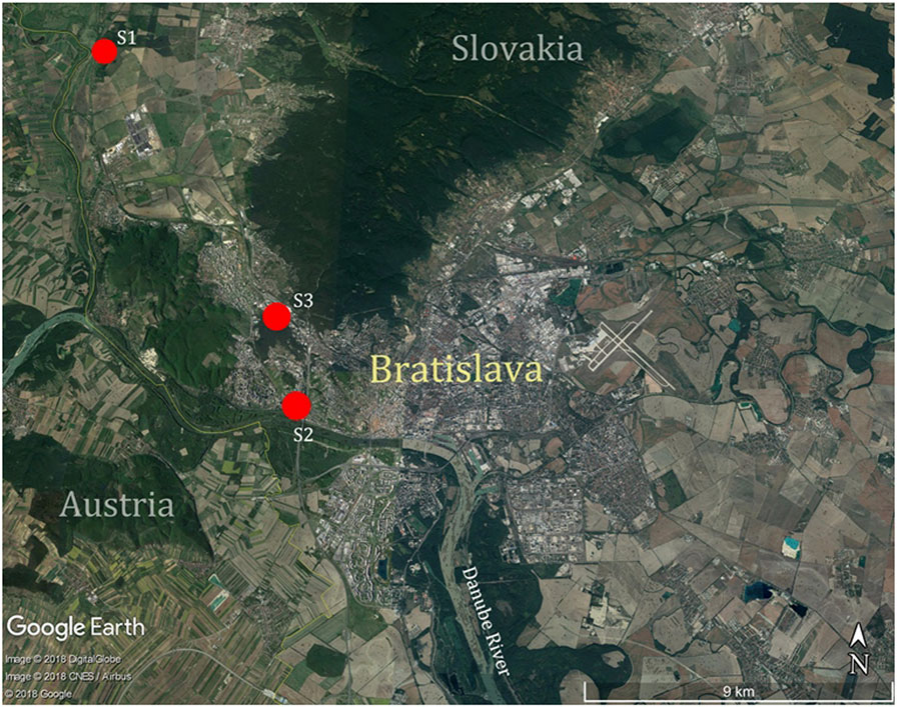
The sampling sites of mosquitoes in two different habitats of Bratislava. (S1: Devínske Jazero settlement, urban-fringe zone; S2: Danube riverbank, residential zone; S3: dog shelter, residential zone). Source: Google Earth

Three localities for mosquito sampling were selected in two different habitats of Bratislava. One of them (Devínske Jazero settlement) was located in an urban-fringe zone, specifically a garden zone, and the other two sampling localities (the Danube riverbank and a dog shelter) were in a residential area in the wider city centre (Fig. 1).

Bratislava is situated in the south-western part of Slovakia at the border with Austria, and it covers an area of 368 km^2^. The site belongs to the Zahorska Lowland geomorphological unit, which is a part of the Vienna Basin in the Carpathian-Pannonian system. Bratislava is located in an area with the warmest and driest climate in Slovakia. The annual average air temperature in this area reaches 10.3 °C, with 2100 h of sunlight annually and a mean annual precipitation of 642 mm [13].

The first studied locality (S1), Devínske Jazero (48°15′43″N, 48°15′43″E) is one of the garden zones of Bratislava. This settlement is situated in the Slovak part of the Ramsar Site along the River Morava. Periodic flooding of the Morava creates a floodplain area of size 12 km^2^. This site is characterised by the largest alluvial meadows of the *Cnidion venosi* communities in central Europe, a floodplain of softwood forests and eutrophic lakes. The occurrence of many migrating birds in this area contributed to its being designated as an Important Bird Area (IBA). This locality is significantly influenced by the Morava and Danube rivers: the alluvial meadows are flooded in spring and then dry up over the summer [13–15].

The second sampled locality (S2) was situated on the Danube riverbank (100 m from the River Danube itself), directly in a highly urbanised area of the city centre (48°08′52.0″N, 17°04′17.5″E). The promenade located on the Danube riverbank serves as a recreational, sport and walking area for city residents and their pets. A highly visited botanical garden and kayak boathouses are located in the close vicinity. The Karloveské Rameno river branch, one of the few free-flowing river branches in the Slovak section of Danube, adjoins with this sampling area. The vegetation is represented by species characteristic for floodplain forests: *Populus nigra* (black poplar), *Ulmus laevis* (European white elm), *Fraximus excelsior* (European ash), *Populus alba* (white poplar) and *Salix alba* (white willow) [16].

The third sampling site (S3) was located in a dog shelter in Bratislava (48°10′25.6″N, 17°03′37.5″E). The site is situated in the wider city centre, surrounded by a fragment of oak-hornbeam forest in the Líščie údolie valley [13]. The forest connects this area with the neighbouring Bratislava zoological garden, which leads to an assumption of high animal host density. In all, a dozen kennels for several hundred dogs are located here.

### Mosquito trapping and identification

Mosquito collection was performed during three sampling periods conducted between April and October of the years 2015, 2016 and 2017 using BG-Sentinel and BG-Mousquetaire traps (Biogents AG, Regensburg, Germany) enriched with carbon dioxide. The collected mosquitoes were stored at -20 °C until their identification. The species richness in the urban-fringe and residential zone was quantified by Menhinick’s diversity index (D) which is the ratio of the number of taxa to the square root of sample size (D = s/√N) where “s” equals the number of different species represented in the sample, and “N” equals the total number of individual organisms in the sample.

Female mosquito individuals were identified by morphological features under a Leica M125 stereomicroscope (Leica Microsystems GmbH, Wetzlar, Germany) according to the identification key by Becker et al. [2]. Collected females were pooled pursuant to sampling site, trapping date and species from a minimum of one to a maximum of 25 individuals per pool and were stored at -20 °C. In addition, molecular methods were used for determination of damaged material and selected individuals belonging to cryptic species from the *Anopheles maculipennis* and *Culex pipiens* complexes.

Individuals selected for molecular determination were placed into 2 ml Eppendorf tubes with 80–100 μl PBS and a 5 mm stainless steel bead (Qiagen, Hilden, Germany) and homogenized in Qiagen TissueLyser (Qiagen) at 30 Hz/6 min. After homogenization, the samples were incubated with 30 μl proteinase K and 180 μl ATL buffer at 56 °C for 3 h. Subsequently, DNA was isolated using the DNeasy Blood & Tissue Kit (Qiagen) following steps in the protocol.

In cases when materials were damaged during trapping, the positive mosquito individuals and pools were verified to the species level by standard polymerase chain reaction (PCR). Fragments of the cytochrome *c* oxidase subunit 1 (*cox*1) gene were amplified using primers MTRN/MTFN designed by Kumar et al. [17]. PCR was carried out under the following thermal conditions: 94 °C for 2 min, followed by 35 cycles at 94 °C for 30 s, 57 °C for 30 s, 72 °C for 30 s and a final extension at 72 °C for 7 min. PCR amplicons were sequenced in both directions by Sanger sequencing. The newly generated sequences were compared with entries available in the GenBank database.

Individuals of morphologically identical members of the *Culex pipiens* complex (18 females) were selected to be identified by two multiplex PCR assays. First, the variations of the nuclear locus in the acetylcholinesterase-2 gene (*ace*2) were examined to differentiate *Cx. pipiens* biotypes from *Culex torrentium*. The primer set ACEpall/ACEpip/ACEtorr/B1246s targeting a 416 bp portion fragment in *Cx. torrentium*, a 478 bp fragment in *Cx. pipiens pallens* and a 610 bp fragment in *Cx. pipiens*; forms were used as previously described by Smith & Fonseca [18] and Zittra et al. [19]. Afterwards, microsatellites of the locus CQ11 were targeted using primers CQ11F/pipCQ11R/molCQ11R according to Bahnck & Fonseca [20] for identification of the *Cx. pipiens* biotypes and their hybrids. Product sizes of 180 bp for *Cx. pipiens* biotype *pipiens* and 240 bp for *Cx. pipiens* biotype *molestus* were expected.

In addition, 117 females from the *Anopheles maculipennis* group were identified by multiplex PCR assay, as described previously by Blažejová et al. [21]. Species-specific primers were used to mark the internal transcribed spacer 2 (ITS2) rRNA regions for members of *An. maculipennis* (*s.l.*), *An. atroparvus*, *An. sacharovi*, *An. melanoon*, *An. labranchiae*, *An. daciae* and *An. messeae*, as designed by Kronefeld et al. [22]. PCR products were visualised by electrophoresis on 1.5% agarose gels. Selected amplicons were purified and sequenced (as is described below).

### Molecular screening for filarioid species

For DNA extraction, mosquitoes were divided into pools. To each mosquito pool 180–800 μl PBS (according to the number of mosquitoes in pool) and a 5 mm stainless bead (Qiagen) were added. Samples were homogenized in Qiagen TissueLyser (Qiagen) at 30 Hz/6 min. After homogenization, 50 μl proteinase K was added to each sample and left to incubate at 56 °C for 24 hours. DNA extraction was provided by DNeasy Blood & Tissue (Qiagen®, Hilden, Germany), according to the manufacturer’s protocol.

The presence of filarioid DNA was detected by standard PCR. Owing to the expectation of a low concentration of parasite DNA, each sample was re-amplified. In each run, a positive sample (DNA isolated from filarioid worm) and a negative control (nuclease-free water) were used. Every single step, including mosquito identification, DNA extraction, master-mix preparation and gel electrophoresis, was performed in a separate place to prevent sample contamination. For the first screening, universal nematode primers COIintF/COIintR, targeting an approximately 650 bp long fragment of the filarioid *cox*1 gene, was used [23]. Positive pools were subsequently examined by species-specific primers for *D. repens* (DR COI-F1/DR COI-R1) and *D. immitis* (DI COI-F1/DI COI-R1) targeting a fragment of the *cox*1 gene, as designed by Rishniw et al. [24], and primer sets StCol 616L/StCol1105L to mark a 730 bp region of the *cox*1 gene and StND4L/StCol747H targeting an 860 bp long fragment of the *cox*1 and *nad*4 genes of members belonging to the genus *Setaria* [25].

Furthermore, the selected amplicons were purified using NucleoSpin® Gel and the PCR Clean-up kit (Macherey-Nagel GmbH & Co. KG, Düren, Germany) and commercially sequenced in both directions by Sanger sequencing. The Fasta format of sequences were manually edited in MEGA 7 and compared by BLAST with sequences available in GenBank. The respective sequences are deposited in the GenBank database.

## Results

### Mosquito species composition and abundance

A total of 6957 female mosquitoes were collected for this study during the sampling period. Different mosquito species (*Aedes vexans*, *Anopheles hyrcanus*, *Anopheles plumbeus*, *Coquillettidia richiardii*, *Culex modestus*, *Culiseta annulata*, *Ochlerotatus caspius* and *Ochlerotatus sticticus*), two groups of morphologically cryptic species (*Anopheles maculipennis* complex and *Culex pipiens* complex) and individuals of *Aedes cinereus*/*geminus* that cannot be identified according their morphological features were trapped (Table 1). Some specimens (*n =* 970) were damaged during the sampling process, and their morphological identification was possible only to the genus level (*Aedes* spp., *Anopheles* spp. and *Culex* spp.). Overall, the most abundant mosquito species was *Ae. vexans* with 38.7% of the total catch, closely followed by unidentified members of the *Cx. pipiens* complex (36.5% of the total catch) and the less numerous, but still plentiful *Ochlerotatus sticticus* species (6.0% of the total catch). Great differences were observed between mosquito abundances in the two monitored habitats of the city. Most of the studied mosquitoes, as many as 6279, were trapped in the urban-fringe zone (sampling site S1), while in the urban area (residential zone), only 678 specimens were collected during the entire sampling period. Also, more mosquito taxa were captured in the urban-fringe area; for instance, *An. hyrcanus*, *Cs. annulata* and *Och. caspius* were collected only at the Devínske Jazero sampling site (S1), though only in a few isolated cases. In contrast, *An. plumbeus* and *Cx. modestus* species were observed only in the residential zone of the city. However, using Menhinick’s diversity index the calculated mosquito richness was higher in the sampling sites of a residential zone (Table 1).

**Table 1:** Mosquito species/complexes collected in an urban-fringe zone (sampling site S1) and a residential zone (sampling localities S2 and S3) of Bratislava, Slovakia. The most abundant species are highlighted in bold

Species/complex	Number of mosquitoes		
	Urban-fringe zone (S1)	Residential zone (S2 + S3)	Total
*Aedes cinereus*/*geminus*	4	1	5
*Aedes vexans*	2661	29	**2690**
*Aedes* spp.	764	2	766
*Anopheles hyrcanus*	1	–	1
*Anopheles maculipennis* complex	126	10	136
*Anopheles messeae*	56	na	56
*Anopheles daciae*	61	na	61
*Anopheles plumbeus*	–	13	13
*Anopheles* spp.	1	–	1
*Coquillettidia richiardii*	15	33	48
*Culex modestus*	–	2	2
*Culex pipiens* complex	2005	534	**2539**
*Cx. pipiens* biotype *pipiens*	2	11	13
*Cx. pipiens* biotype *molestus*	–	5	5
*Culex* spp.	192	10	202
*Culiseta annulata*	1	–	1
*Ochlerotatus caspius*	3	–	3
*Ochlerotatus sticticus*	387	27	**414**
*Ochlerotatus* spp.	–	1	1
Total	6279 (D = 0.19)	678 (D = 0.50)	6957

*Abbreviations*: S1, Devínske Jazero settlement; S2, Danube riverbank; S3, Dog shelter; na, molecular identification was not performed from this locality; D, Menhinick’s diversity index

The designated material from the cryptic groups were analysed based on PCR assays and sequencing. The assay, which targets the locus for *ace*2, discriminated only *Culex pipiens* biotypes. *Culex torrentium* was not detected in our study. From the examined mosquitoes (*n* = 18), 13 individuals were identified as the *Cx. pipiens* biotype *pipiens* and 5 mosquitoes as the *Cx. pipiens* biotype *molestus*. A multiplex PCR for species-specific determination of members of the *An. maculipennis* complex (*n* = 117) revealed two species, *An. daciae* (*n =* 56) and *An. messeae* (*n* = 61) (Table 1).

### Detection of filarioid nematodes

Four hundred and fifty-one mosquito pools from the urban-fringe zone and 159 pools from the residential zone were processed for molecular screening for filarioid nematodes. Altogether, fragments of filarioids *cox*1 gene were detected in 26 pools (4.26%). Consequential analyses showed *D. repens* was identified as the predominant filarioid species: it was detected in 22 samples (3.61%). In contrast, mono-infection of *D. immitis* was detected in only two pools (0.33%), and in two samples a mixed infection of *D. repens* and *D. immitis* was confirmed (0.33%). DNA of *Setaria* species was not detected in any of the pools examined.

Most of the positive mosquito pools (*n* = 24) had their origin in the floodplain area situated on the outskirts of the city, in the Devínske Jazero settlement, with a relative prevalence of 5.32%. *Dirofilaria repens* (*n* = 22) was found predominately in this area, but a single case of *D. immitis* and one mixed infection were also detected here. In comparison, only two mosquito pools were shown to be positive in the residential zone of Bratislava (1.26%): in one of them only *D. immitis* DNA was confirmed, and in the second both *D. immitis* and *D. repens* were detected (Table 2).

**Table 2:** *Dirofilaria* species detected in mosquitoes collected from different urban habitats of Bratislava, Slovakia

*Dirofilaria* spp.	Number of positive mosquito pools for *Dirofilaria* spp. (%)		
	Urban-fringe zone (S1) (*n* = 451)	Residential zone (S2 + S3) (*n* = 159)	Total (*n* = 610)
*D. repens*	22 (4.88)	–	22 (3.61)
*D. immitis*	1 (0.22)	1 (0.63)	2 (0.33)
*D. repens* + *D. immitis*	1 (0.22)	1 (0.63)	2 (0.33)
Total	24 (5.32)	2 (1.26)	26 (4.26)

*Abbreviations*: S1, Devínske Jazero settlement; S2, Danube riverbank; S3, Dog shelter; *n*, number of examined mosquito pools

Regarding vector species, *D. repens* was identified primarily in *Ae. vexans* (9 pools positive) and unidentified members of the *Culex pipiens* complex (7 pools positive). In addition, 4 pools of *An. messeae* and 2 pools of the unidentified *An. maculipennis* complex were positive for *D. repens*. *Dirofilaria immitis* DNA was detected only in *Cq. richiardii* and the *Cx. pipiens* biotype *pipiens.* One pool of *Ae. vexans* and one of *Och. sticticus* were shown to be positive for both *D. repens* and *D. immitis* (Table 3).

**Table 3:** Occurrence of *Dirofilaria* spp. in various mosquito species/complexes collected in Bratislava, Slovakia

Mosquito species/complex	Number of positive mosquito pools for *Dirofilaria* spp. (%)			
	*D. repens*	*D. immitis*	*D. repens* + *D. immitis*	Total
*Aedes vexans* (*n* = 138)	9 (6.52)	–	1 (0.72)	10 (7.25)
*Anopheles maculipennis* complex (*n* = 28)	2 (7.14)	–	–	2 (7.14)
*Anopheles messeae* (*n* = 61)	4 (6.56)	–	–	4 (6.56)
*Coquillettidia richiardii* (*n* = 26)	–	1 (3.85)	–	1 (3.85)
*Culex pipiens* complex (*n* = 187)	7 (3.74)	–	–	7 (3.74)
*Cx. pipiens* biotype *pipiens* (*n* = 6)	–	1 (16.67)	–	1 (16.67)
*Ochlerotatus sticticus* (*n* = 41)	–	–	1 (2.44)	1 (2.44)
Total (*n* = 610)	22 (3.61)	2 (0.33)	2 (0.33)	26 (4.24)

*Abbreviations*: *n*, number of examined mosquito pools

Of the selected *cox*1 locus, a 111 bp long overlapped fragment of *D. repens* isolated from *Ae. vexans* and a 211 bp long fragment of *D. immitis* isolated from *Och. sticticus* revealed 100% similarity with the sequences obtained from Slovak dogs (KC985239 and KC985240, respectively). Both nucleotide sequences of *D. repens* and *D. immitis cox*1 gene were deposited in the GenBank database under the accession numbers MG787424 and MG787425.

## Discussion

Currently, mosquitoes are at the centre of medical and veterinary research because of their importance as vectors of a wide range of viral and parasitic infections [2, 26–28]. In Slovakia, concern over mosquitoes has risen during the last decade. Still, research in this field is very rare, and published data are both very limited and sporadic. Moreover, the results of numerous studies have confirmed the emergence of mosquito-borne infections as becoming especially associated with urban environments and anthropization [7, 29]. Therefore, our study presents an initial survey focused on species composition and the abundance of mosquitoes inhabiting Bratislava, as well as the first molecular screening for mosquito-transmitted filarioid nematodes in Slovakia. The studied area is unique from several points of view. Bratislava is the only capital city which neighbours directly with two other countries, Austria and Hungary, and considering flight activity of some mosquito species, this fact puts a transboundary output of our study [2, 13]. Regarding habitat and ecological conditions, Bratislava is located in a very warm and dry area which favours incubation and development of *Dirofilaria* in mosquitoes [6]. In addition, the city is located along both sides of River Danube and several times in the past; the rising river has caused extensive floods directly in the city centre, followed by mosquito outbreaks [30, 31].

Almost 7000 mosquitoes were collected during the study, and the mosquito species recorded here are commonly widespread in central Europe. Together, 12 different mosquito taxa (*Ae. vexans*, *An. hyrcanus*, *An. messeae*, *An. daciae*, *An. plumbeus*, *Cq. richiardii*, *Cx. modestus*, *Cx. pipiens* biotype *pipiens*, *Cx. pipiens* biotype *molestus*, *Cs. annulata*, *Och. caspius* and *Och. sticticus*) were distinguished, in addition to unidentified individuals of *Ae. cinereus*/*geminus*. Our sampling sites were selected in two different habitats: a highly urbanized residential area of the wider city centre (S2 + S3) and an urban-fringe habitat represented by a garden zone of the city (S1). The type of habitat and its individual conditions influenced the mosquito captures and the community composition in separate trapping areas of Bratislava. According to the number of trapped mosquitoes, it is obvious that the more natural habitat creates better conditions for mosquito biology [7]. The number of mosquitoes collected from the Devínske Jazero sampling site in the urban-fringe zone was more than nine-times higher than that of the residential zone in the city centre.

It is not an unexpected finding that the most abundant species trapped were *Ae. vexans* and members of the *Cx. pipiens* complex. *Aedes vexans* is a cosmopolitan species, typical for floodplain areas, and the floodplain area of the River Morava on the outskirts of Bratislava seems to be a very suitable environment for this species. *Aedes vexans* was also found to be the most abundant mosquito species in the Lower Austria Province directly neighbouring the Bratislava region with a large floodplain habitat is very similar to that of Devínske Jazero settlement (sampling site S1 in our study) [32]. The same results with *Ae. vexans* being the most frequent species, were also reported from two other surrounding countries: the Czech Republic and Hungary [33, 34].

The next highly abundant mosquito species recorded within the present study belong to the *Cx. pipiens* complex. To our knowledge, molecular identification of the *Cx. pipiens* complex in Slovakia was performed for the first time, and two biotypes were found: the ornithophilic form *pipiens*, which plays only a minor role as a virus vector, and the medically important anthropophilic and mammophilic form *molestus*, which is the main vector of West Nile virus (WNV) [2]. The biotype *pipiens* was more abundant than the biotype *molestus* in our study*.* The same results were previously observed in Austria and in Germany, where the *molestus* biotype were absent in the entire northern and eastern parts of the country [19, 35]. In our study, the *molestus* biotype was not detected in the urban-fringe environment, which corresponded with its habitat preferences as a typical urban type [2]. Our preliminary results bring important data, because both biotypes were captured in the same area of an urban environment in this study. The hybridization of these forms was observed in areas of their co-existence, and their hybrids may serve as important bridge vectors of WNV from birds to humans [19]. In a period of frequent WNV outbreaks in several parts of Europe, this finding is greatly disturbing and indicates a risk for human health, especially in urban areas with high density of citizens [36, 37]. The role of dogs as a reservoir of WNV is improbable due to low levels of viremia, but they can serve as sentinels of the infection [38]. In Slovakia, the circulation of WNV in birds was also confirmed recently [39], but extensive research for identifying the real potential of *pipiens*/*molestus* hybrid occurrence should be done. Two female specimens of another principal vector of WNV in Europe, *Cx. modestus*, were trapped only on the Danube riverbank in the residential zone. The neighbouring Karloveské Rameno river branch, overgrown by reed beds, should be preferred by this species, and its occurrence on the Danube promenade should be expected in the summer months.

Only recently, the first comprehensive study on species distribution within the *An. maculipennis* complex was performed, and it confirmed *An. daciae* as a new species in Slovakia [21]. Molecular identification of the *maculipennis* complex was carried out only at the Devínske Jazero sampling site, in an urban-fringe zone where two species, *An. daciae* and *An. messeae*, were caught. In contrast, several years ago, no individuals of *An. maculipennis* (*s.l.*) were detected in this locality [40]. The density of both collected species was relatively equal (61 and 56 females, respectively). While *An. daciae* has been reported for Slovakia only recently, the occurrence of *An. messeae* was already recognised in 1955, when this species was confirmed as the main vector of malaria in this country [41]. Two other species of the genus *Anopheles* were found within the present study, *An. hyrcanus* and *An. plumbeus*, both with a very low abundance. *Anopheles hyrcanus* was trapped in the urban-fringe zone at Devínske Jazero settlement, in the same area where the species was observed for the first time in Slovakia several years ago [42, 43]. This species was also confirmed in an adjacent Lower Austria Province and in other neighbouring countries, Hungary and the Czech Republic, where *An. hyrcanus* was reported quite recently, in 2003 and 2008, respectively [32, 43, 44]. This thermophilic mosquito is widely distributed in the Mediterranean area and represents an invasive species in central Europe. Moreover, today *An. hyrcanus* is considered an important potential vector for human and avian malaria [45], and at present, when climate change is influencing species distribution, the occurrence of a stable population of *An. hyrcanus* in the warmest area of Slovakia may represent a serious risk. *Anopheles plumbeus* was trapped only in the residential zone of Bratislava. Although this mosquito species is predominantly associated with natural grassland and agricultural areas, several studies have pointed to its increasing occurrence in urban environments [46]. Taking into account that *An. plumbeus* acts as a bridge vector of tropical malaria parasite as well as WNV, the observed inclination towards urban environments might represent an important public health problem in the future [46, 47].

Another mosquito species, *Cq. richiardii*, was also more frequent at the sampling sites located in the residential part of the city. Its anthropophilic behaviour is known even in areas surrounded by fresh waters, in the same conditions as on the Danube riverbank. In neighbouring eastern Austria, *Cq. richiardii* was the most abundant species, with 31.4% of the total catch, and was positively associated with slightly elevated Danube water levels and negatively associated with precipitation [32].

Two *Ochlerotatus* species were identified during this study: three specimens of *Och. caspius* and the more numerous *Och. sticticus* predominantly occurred in Devínske Jazero settlement in the urban-fringe zone. *Ochlerotatus sticticus* was also found to be highly abundant in the Lower Austria Province and showed a strongly positive relation to elevated Danube water levels and excessive periods of sunshine [32].

Molecular analysis of the trapped female mosquitoes for the presence of *Dirofilaria* DNA revealed the expected dominance of *D. repens* in Slovakia. This finding correlates with the results of the extensive epidemiological study provided by Miterpáková et al. [9], where a majority of dogs in Slovakia were infected with *D. repens*. Regarding *D. immitis*, using blood-screening methods (Knott test and PCR) only ten dogs with heartworm disease were recorded in Slovakia within the previous ten years (in nine cases a mixed infection with *D. repens* and in one dog only *D. immitis* was confirmed). However, the following serologic survey revealed that *D. immitis* could be expanded more widely concerning occult infections in certain endemic areas [48]. This hypothesis is also supported herein by the presented results, as *D. immitis* has been detected in mosquitoes trapped in both studied habitats.

Only one study regarding mosquito investigation for dirofilarial DNA presence has thus far been carried out in Slovakia [12]. This research based in the Košice region, in the eastern part of Slovakia, confirmed *Ae. vexans* as a vector for both *D. repens* and *D. immitis*. However, a large variety of mosquito species can serve as vectors for *Dirofilaria* spp. [2]. In the presented study, five different species (*Ae. vexans*, *An. messeae*, *Cq. richiardii*, *Cx. pipiens* biotype *pipiens* and *Och. sticticus*) and a few unidentified members from the *Cx. pipiens* and *Anopheles maculipennis* complexes were confirmed as potential vectors of *Dirofilaria* spp. in Slovakia. The majority of positive pools was detected among the *Ae. vexans* and the *Cx. pipiens* complex, two of the most abundant mosquito taxa collected in this study. *Aedes vexans*, the floodplain mosquito species, is abundant in the summer months, in the period when the peak activity of microfilaria fluctuation was identified in dogs from Slovakia [2, 9]. This most abundant and one of the frequently infected species in Europe (Italy, Germany, the Czech Republic, Hungary, Serbia, Romania) plays a key role in the spread of *Dirofilaria* spp. [34, 49–53].

Due to late establishment of the new molecular differentiation of cryptic groups, only limited data are available on filarioid occurrence in these mosquitoes. In our study, only *An. messeae* was found to be positive for *D. repens*, while a study in Germany by Kronefeld et al. [50] confirmed *D. repens* only in *An. daciae*. To the best of our knowledge, these are the only two screenings for *Dirofilaria* parasites among species identified within the *An. maculipennis* group in Europe.

Two mosquito taxa were found to be positive solely for *D. immitis* in our study. Surprisingly, the heartworm DNA was detected in the ornithophilic form *Cx. pipiens* biotype *pipiens* which prefers birds as blood hosts. Recently, the same results were obtained in Spain, where *D. immitis* was found in the head and thorax of the *pipiens* biotype. Accordingly, it could be suggested that the *Cx. pipiens* biotype *pipiens* might be a competent vector for canine heartworm transmission [54] and these findings may bring a new insight into the understanding of the low prevalence of *D. immitis* infection in Slovakia. It is likely that this occasionally anthropophilic and mammophilic feeder is not able to infect a high number of carnivore hosts. On the other hand, *D. immitis* solitary or together with *D. repens*, was also detected in pools of animal- and human-feeding mosquito species: *Ae. vexans*, *Och. sticticus* and *Cq. richiardi*. Although mosquito field studies are not able to specify the effectiveness of parasite transmission, it is clear that some unidentified differences exist in *D. immitis*-vector specificity [55]. Several previous studies have declared genetic and morphological differences responsible for the susceptibility or resistance of various mosquito species to *Dirofilaria* parasites [56, 57], but little is known about interactions between both dirofilarial species when they coincide in the same mosquito host [6]. In our study, only two mosquito species (*Ae. vexans* and *Och. sticticus*) hosted both *Dirofilaria* spp.

The relative prevalence of dirofilariosis detected in our study was shown to be higher than in neighbouring Austria, the Czech Republic and Poland. Furthermore, only *D. repens* was found in mosquitoes from these countries [50, 58, 59]. On the other hand, in mosquitoes from Hungary, *D. immitis* was also confirmed, and the relative prevalence of *D. repens* was higher when compared to Slovakia [34].

It has been suggested that a high density of suitable definitive hosts should be related with higher abundance of dirofilariosis [60]. However, the majority of *Dirofilaria*-positive mosquito pools came from the Devínske Jazero settlement, the more natural, less urbanised area of Bratislava. Although mosquito species richness quantified by Menhinick’s diversity index was higher in the residential zone, the garden zone in Devínske Jazero and an adjacent floodplain area of the River Morava create proper conditions for mosquito and *Dirofilaria* development. This is evidenced by large number of captured mosquitoes, relatively high species richness and also by their high positivity for *Dirofilaria* spp.

On the contrary, in the city centre, dirofilarial DNA was identified in only 1.26% of analysed mosquito pools. Moreover, the number of trapped mosquitoes was much lower despite the use of the same sampling method. On the other hand, monoinfection with *D. immitis* was detected only in the urban habitat. In addition, in recent years extensive floods followed by mosquito outbreaks have frequently occurred directly in the city centre of Bratislava, which creates a potential risk for increasing the abundance of dirofilariosis not only in dogs but also in humans.

## Conclusions

This study brings important data concerning mosquito species abundance and composition in urban area of Bratislava, Slovak capital city. In addition, primary molecular analyses within the *Cx. pipiens* complex in Slovakia were performed, and the co-occurrence of two biotypes were identified in the same environment: the *Cx. pipiens* biotype *pipiens* and the *Cx. pipiens* biotype *molestus*. Further investigation of mosquito material revealed 4.26% relative prevalence of *Dirofilaria* spp., whereby both species, *D. repens* and *D. immitis*, were identified. *Dirofilaria* spp. were detected for the first time in Slovakia in mosquitoes other than *Ae. vexans*, i.e. *D. repens* in *Anopheles messeae* and unidentified members of *An. maculipennis* and *Cx. pipiens* complexes, and *D. immitis* in *Coquillettidia richiardii* and *Cx. pipiens* biotype *pipiens.* Both dirofilarial species in were confirmed in *Och. sticticus*. The more natural habitat of the urban-fringe zone creates appropriate conditions for mosquito biology. Also, most of the positive pools came from this area, though the occurrence of potential definitive hosts was more abundant in the urban zone. Accordingly, the suitable conditions for vectors’ survival would represent the main risk factor for dirofilariosis transmission.

## Abbreviations

PCR: Polymerase chain reaction
DNA: Deoxyribonucleic acid
*cox*1: Cytochrome *c* oxidase subunit 1
IBA: Important Bird Area
ace2: Acetylcholinesterase-2 gene
CQ11: Microsatellite of the locus CQ11
ITS2: Internal transcribed spacer 2
rRNA: Ribosomal ribonucleic acid
PBS: Phosphate-buffered saline
*nad*4 gene: Reduced nicotinamide adenine dinucleotide of the dehydrogenase subunit 4
BLAST: Basic Local Alignment Search Tool
